# Validation of the German Montreal-Cognitive-Assessment-H for hearing-impaired

**DOI:** 10.3389/fnagi.2023.1209385

**Published:** 2023-07-19

**Authors:** Christiane Völter, Hannah Fricke, Sarah Faour, Gero Lueg, Ziad S. Nasreddine, Lisa Götze, Piers Dawes

**Affiliations:** ^1^Department of Otorhinolaryngology, Head and Neck Surgery, Catholic Hospital Bochum, Ruhr University Bochum, Bochum, Germany; ^2^Department of Geriatric Medicine, Marien Hospital Herne, Ruhr University Bochum, Herne, Germany; ^3^MoCA Clinic and Institute, Greenfield Park, QC, Canada; ^4^Centre for Hearing Research (CHEAR), School of Health and Rehabilitation Sciences, University of Queensland, Brisbane, QLD, Australia

**Keywords:** MoCA-H, cognitive screening, dementia, mild cognitive impairment, hearing loss

## Abstract

**Background:**

Hearing loss and dementia are highly prevalent in older age and often co-occur. Most neurocognitive screening tests are auditory-based, and performance can be affected by hearing loss. To address the need for a cognitive screening test suitable for people with hearing loss, a visual version of the Montreal-Cognitive-Assessment was developed and recently validated in English (MoCA-H), with good sensitivity and specificity for identifying cases of dementia. As the MoCA is known to perform differently across languages, revalidation of the German MoCA-H was necessary. The aim of the present study was to assess the diagnostic accuracy of the German MoCA-H among those with normal cognition, mild cognitive impairment (MCI) and dementia and to determine an appropriate performance cut- off.

**Materials and methods:**

A total of 346 participants aged 60–97 years (M = 77.18, SD = 9.56) were included; 160 were cognitively healthy, 79 with MCI and 107 were living with dementia based on the GPCOG and a detailed medical questionnaire as well as a comprehensive examination by a neurologist in case of cognitive impairment. Performance cut-offs for normal cognition, MCI and dementia were estimated for the MoCA-H score and z-scores using the English MoCA-H cut-off, the balanced cut-off and the Youden’s Index.

**Results:**

A mean score of 25.49 (SD = 3.01) points in the German MoCA-H was achieved in cognitively healthy participants, 20.08 (SD = 2.29) in the MCI and 15.80 (SD = 3.85) in the dementia group. The optimum cut-off for the detection of dementia was ≤21 points with a sensitivity of 96.3% and a specificity of 90%. In the MCI group, a cut-off range between 22 and 24 points is proposed to increase diagnostic accuracy to a sensitivity and specificity of 97.5 and 90%, respectively.

**Conclusion:**

The German MoCA-H seems to be a sensitive screening test for MCI and dementia and should replace commonly used auditory-based cognitive screening tests in older adults. The choice of a cut-off range might help to better reflect the difficulty in clinical reality in detecting MCI. However, screening test batteries cannot replace a comprehensive cognitive evaluation.

## Introduction

Hearing loss and dementia are among the most common chronic diseases in older age and frequently co-occur (World Health Organization [WHO], 2020, 2021; McDonough et al., 2021). By 2050 up to 900 million people will be living with hearing loss (World Health Organization [WHO], 2018) and 152.8 million with dementia (Nichols, 2022). In a German sample, dementia cases increased by 0.23% with an increasing prevalence of hearing impairment by 1 SD (Teipel et al., 2015), and an increased risk of cognitive decline was described in case of a bilateral hearing loss with a hazard ratio of 1.43 (Fritze et al., 2016). Hearing loss often remains undetected (Ramage-Morin et al., 2019). Likewise, subjective cognitive complaints do not reliably predict cognitive impairment (Edmonds et al., 2018).

But, people with hearing loss have a higher risk of dementia (Livingston et al., 2020) and cognitive impairment is associated with a higher risk of anxiety, of depression (Mirza et al., 2017 ), and of functional impairment (Brown et al., 2011), loss of independence (McLaughlin et al., 2010), institutionalization (Eska et al., 2013), delirium (Tsui et al., 2022) and mortality (Diwell et al., 2018). Therefore it is an imperative to reliably detect cases of cognitive impairment. Due to the high comorbidity of hearing loss and cognitive impairment (Lin et al., 2013; Teipel et al., 2015; Fritze et al., 2016; Huang et al., 2023; Tamblay et al., 2023), cognitive screening has gained increasing awareness beyond geriatric or psychiatric settings (Mordenfeld et al., 2020; Susano et al., 2020; Canavan and O’Donnell, 2022; Jammula et al., 2022; Tsui et al., 2022) including ENT departments and audiology (Mosnier et al., 2018; Humes, 2020; Sanders et al., 2021; Völter et al., 2022).

Therefore, the development and evaluation of tests for cognitive impairment for people with hearing loss are required to enable early detection as well as timely care and clinical intervention.

However, most cognitive screening tests are auditory-based and require good hearing. Hearing loss or simulated hearing loss leads to a false positive diagnosis of dementia or an overestimation of the actual cognitive impairment (Dupuis et al., 2015; Pye et al., 2017; Füllgrabe, 2020; Völter et al., 2020). For example, simulation of a moderate hearing loss on performance of the Mini Mental Status Examination [MMSE, a similar test to the MoCA (Folstein et al., 1975)] resulted in performance within the ‘dementia’ range among college students with normal cognition (Jorgensen et al., 2016). To mitigate the effects of hearing loss on performance, several attempts were made to adjust cognitive screening tests for people with hearing loss (Pye et al., 2017). Tests were typically adjusted by either i) deleting hearing-depended spoken items or ii) presenting the test in visual form. The problem with the former is that deleting items may have an adverse effect on the reliability of the test (Al-Yawer et al., 2019). The problem with the latter is that changing the modality of the test alters the cognitive demands of the test. One must therefore re-validate a test that has been transposed to visual format with respect to identification of cognitive impairment.

The MoCA is a commonly used, freely available cognitive screening test that has been translated into more than 100 languages (Nasreddine et al., 2005) showing a better sensitivity especially in detecting MCI than other test batteries such as the MMSE (Jia et al., 2021). Around one third of the MoCA items are spoken (Völter et al., 2020). Dawes et al. developed and validated a visually presented version of the MoCA for people with hearing loss (MoCA-H) which was translated into German by forward and backward translation as proposed by Cha et al. (2007), Völter and Götze (2021). In addition to providing written instructions and items, this adaptation also included the replacement of two auditory tasks from the original MoCA (sentence repetition and attention) by alternative items. The MoCA-H was conceived to assess the same cognitive domains with a similar level of difficulty as the original MoCA (Dawes et al., 2019).

In a validation sample of people with hearing loss, including 76 with normal cognition and 83 with dementia, a cut-off of ≤24 yielded high sensitivity and specificity (92.8 and 90.8%, respectively) (Dawes et al., 2023). One strength of the MoCA is the ability to discriminate mild cognitive impairment (MCI) from normal cognition (Nasreddine et al., 2005). Dawes et al. (2023) only established performance of the MoCA-H with respect to identification of dementia.

The performance cut-offs derived by Dawes et al. (2023) for the English MoCA-H may not be optimal for translations of the MoCA-H in other languages. Cultural or linguistic factors have been found to impact upon performance of the MoCA (Ng et al., 2018; Theocharous et al., 2023). Validation studies with the original MoCA have resulted in different estimates of accuracy and optimal cut-offs varied between different languages (O’Driscoll and Shaikh, 2017; Carson et al., 2018). In a previous study we developed normative data for the German-language MoCA-H with people with normal cognition using z-scores and taking age, education and sex into account (Völter et al., 2022). We showed that people with hearing loss performed worse than those with normal hearing on spoken items from the standard MoCA, but there was no difference in performance on the novel visually presented items from the MoCA-H.

The aim of the present study was to determine suitable cut-offs for the German MoCA-H with respect to identification of both MCI and dementia versus normal cognition. We hypothesize that the MoCA-H is a sensitive screening test for MCI and dementia, but that cut-offs and diagnostic accuracy may differ from the English version.

## Materials and methods

### Participants

Participants met the following inclusion criteria: (1) age ≥60, (2) education level ≥7 years, (3) fluent in written and spoken German language as assessed during the recruitment procedure, (4) normal or corrected near visual acuity of ≤0.3 logMAR, (5) GDS-15 (Geriatric Depression Scale – 15) in the normal range (Yesavage et al., 1982), and (6) provided written informed consent. A severe neurological or psychiatric disease, a severe motor disorder as well as the inability to read or a prelingual deafness and acute infectious disease, a delirium, prior operative procedures or current medication with psychoactive drugs that might interfere with testing were exclusion criteria.

To be included in the normal cognition group, participants had to achieve either a score of 9 in the GPCOG [General Practitioner Assessment of Cognition, Brodaty et al. (2002)] patient interview, or a score between 5 and 8 in the GPCOG in combination with a score of 4–6 points in the additional GPCOG informant questionnaire. In addition, there should be no hint for cognitive impairment in the medical history.

The diagnosis of dementia and mild cognitive impairment (MCI) was based on the German S3 guideline on the diagnosis and treatment of dementia (Deuschl and Maier, 2016) by an experienced neurologist based on a comprehensive evaluation and the patient’s records. A score of ≤4 points in the GPCOG interview or a score between 5 and 8 in combination with the GPCOG informant’s questionnaire scoring ≤3 was required. Furthermore, a detailed clinical interview of the participant and a relative, if available, and a review of the medical history and the current medication were done. If required, the diagnostic assessment included a brain scan and laboratory diagnostics. Dementia was separated from MCI based on the functional impact of cognitive difficulties in everyday life, which is the cardinal diagnostic criteria for dementia and was recorded by the participant or a relative and the daily routine in the hospital. Participants with delirium or infection were excluded.

### Procedures

Visual acuity was examined using a near vision panel. All participants underwent audiometric testing by pure tone audiometry at 0.5, 1, 2, and 4 kHz for each ear separately using headphones. 4-pure-tone-average (4PTA) was calculated and hearing loss was grouped according to the WHO definition (World Health Organization [WHO], 1991). The GDS-15 questionnaire was administered to identify depressive symptoms. GPCOG testing was done with hearing devices (e.g., hearing aids or cochlear implants, if used) and MoCA-H testing without hearing devices. After at least 4 weeks a retest of the MoCA-H was conducted in 166 participants. To determine the diagnostic accuracy of the MoCA-H defined by the AUC with a marginal error of 0.05, a confidence level of 95% and an estimated effect size (predicted AUC) of 0.85, 151 participants were required for each group (i.e., normal cognition and cognitive impairment) (Hajian-Tilaki, 2014).

### Statistical analysis

Descriptive statistics, chi-squared tests, t-tests and ANOVAs were used to describe sociodemographic, audiological and cognitive data for the three groups (NC, MCI, and D). Internal consistency of the MoCA-H was calculated using reliability analysis and retest reliability was determined by Pearson correlation of the MoCA-H total scores at both measurement points.

Diagnostic accuracy was determined for NC versus the total cognitively impaired group (MCI + dementia group) and separately for NC versus MCI and NC versus dementia as well as for MCI versus dementia. Then cut-offs were calculated for the MoCA-H raw score, the z-score and the MoCA-H score after adjustment for demographic factors using the balanced cut-off method where sensitivity and specificity are as equal as possible, and for the Youden’s Index where sensitivity + specificity −1 reaches its maximum. Further, a multivariable regression analysis was done to determine whether demographic data impacted the MoCA-H score, and whether adjusting for age and education improved sensitivity and specificity. The resulting area under the curve (AUC) was compared using a two-sided significance test for correlated ROC curves. The McNemar-Test was used to compare the correct classification rates of the MoCA-H cut-offs presented in the present manuscript with the English MoCA-H cut-offs according to Dawes et al. (2023). Furthermore, we plotted sensitivity based on the MCI group against specificity based on the NC group. To obtain a sensitivity and specificity of ≥90%, two new cut-offs with a cut-off range were set.

Analyses were conducted using the statistical program SPSS (Version 28) and Rstudio (2021.09.1). Confidence interval was set at 95% and statistical significance was defined as *p* < 0.05.

## Results

### Demographics

346 participants aged 60–97 years (M = 77.18, SD = 9.56) were included: 160 had normal cognition, 79 had MCI and 107 were living with dementia. Mean GPCOG score for cognitively healthy individuals was 8.08 (SD 1.21) and 3.15 (SD 2.01) for the group of participants with mental impairment [dementia 2.48 (SD 1.89) and MCI 4.06 (SD 1.81)]. 182 participants were suffering from moderate to profound hearing loss (4PTA on the better hearing ear ≥40 dB, WHO 2, 3 and 4), 164 participants had normal hearing or were only mild hearing-impaired (4PTA on the better hearing ear <40 dB, WHO 0 and 1). Mean level of depressive symptoms was 2.63 (SD 2.46) for healthy and 4.26 (SD 2.03) for cognitively impaired individuals [dementia 4.28 (SD 1.90) and MCI 4.23 (SD 2.20)]. None of the participants had major depression according to the GDS-15 screening.

There was no significant difference in age (*p* = 0.09) or in educational years (*p* = 0.95) between MCI and dementia participants, while both cognitively impaired groups were significantly older and less educated than the NC group [*F* (2.343) = 121.26, *F* (2.343) = 47.76, *p* < 0.001]. There was a significant group difference in terms of gender distribution with significantly fewer men in the normal cognition group than in the cognitively impaired participants [χ^2^ (1) = 26.29, *p* < 0.001] (Table 1).

**TABLE 1:** Demographic data of the study group.

	Cognitively healthy (*n* = 160)	Cognitively impaired (*n* = 186) (Dementia *n* = 107/ MCI *n* = 79)
	***M* (SD)**	***M* (SD)**
Female (%)	43.1	Total 70.4 Dementia 68.2/MCI 73.4
Age (years)	70.61 (8.29)	Total 82.84 (6.47) Dementia 83.71 (6.28)/MCI 81.67 (6.58)
Education (years)	13.51 (3.29)	Total 10.66 (2.08) Dementia 10.62 (2.24)/MCI 10.71 (1.86)
4PTA (dB)	43.45 (30.64)	Total 43.73 (13.92) Dementia 44.88 (12.83)/MCI 42.17 (15.22)
MoCA-H total score	25.49 (3.01)	Total 17.62 (3.90) Dementia 15.80 (3.85)/MCI 20.08 (2.29)

**FIGURE 1:**
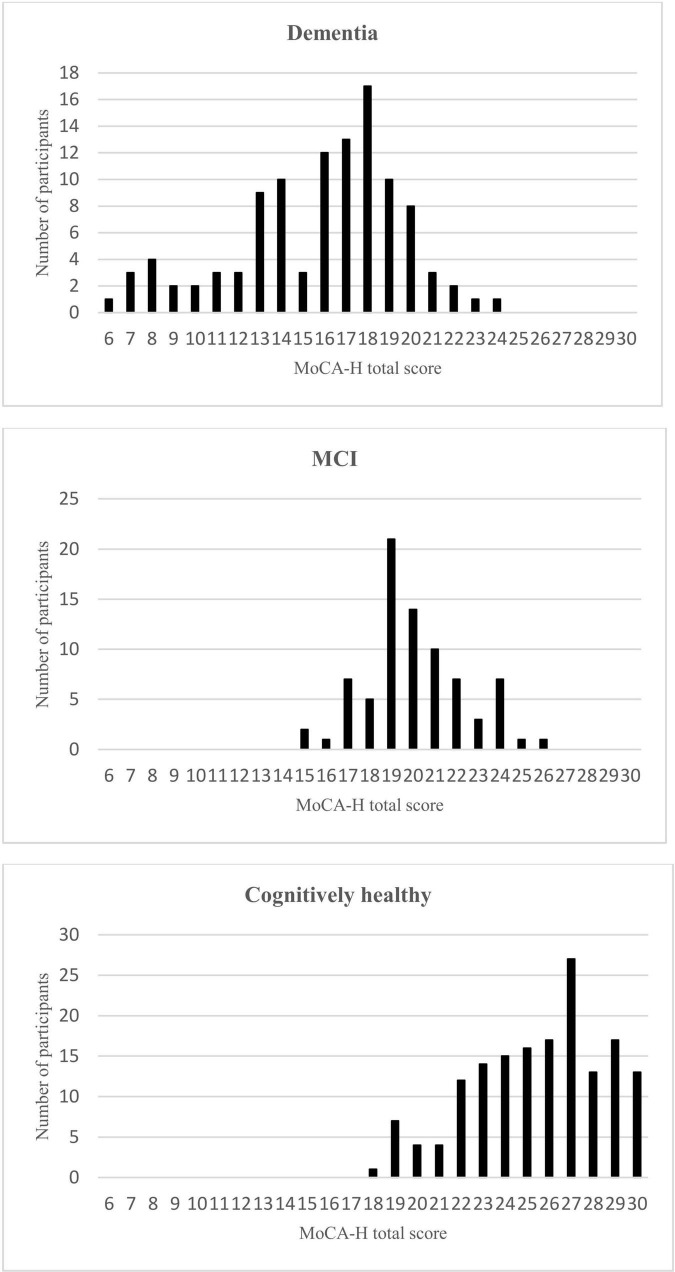
MoCA-H total scores for the Dementia, MCI, and cognitively healthy subjects.

All groups differed in the total score of the MoCA-H, with the normal cognition group scoring significantly higher than those with MCI or dementia [*F* (2.343) = 308.89, *p* < 0.001]. The MCI group had a significantly higher average score than the dementia group (*p* < 0.001) (Table 1). Figure 1 shows the MoCA-H scores for each group separately.

### Retest-reliability and internal consistency

Retesting was done in 166 participants on average 84.02 (SD 47.92) days after the first testing. Retest reliability was high with a Pearson correlation of .937. Internal consistency was good with a Crohnbachs α of 0.8.

### Diagnostic accuracy of the MoCA-H

**FIGURE 2:**
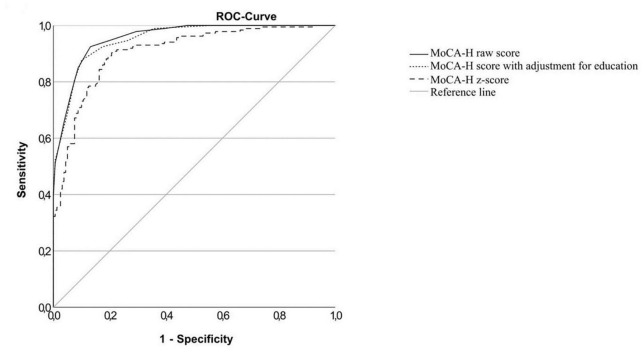
ROC-curves of the MoCA-H for the cognitively impaired group in total (MCI + Dementia) and the cognitively healthy group.

**FIGURE 3:**
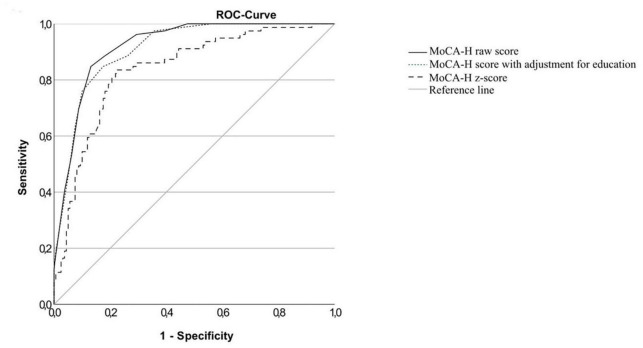
ROC-curves of the MoCA-H for the MCI group and the cognitively healthy group.

**FIGURE 4:**
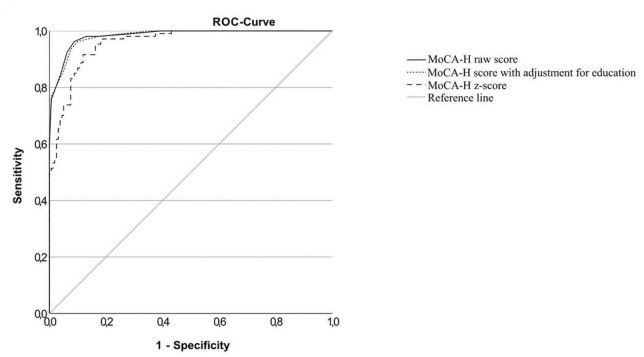
ROC-curves of the MoCA-H for the dementia group and the cognitively healthy group.

**FIGURE 5:**
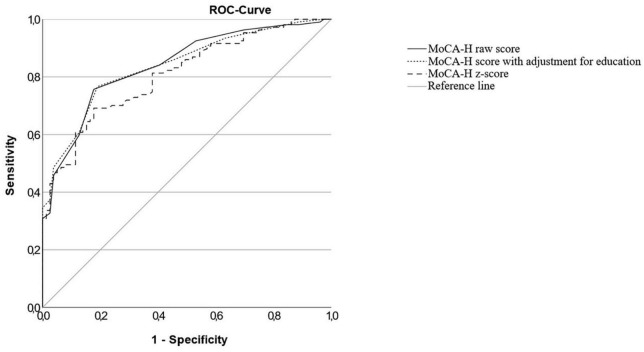
ROC-curves of the MoCA-H for the MCI and the dementia group.

The ROC curves for the total cognitive impaired sample (IC), the MCI sample (MCI) and the dementia sample (D) versus the cognitively normal sample and the MCI sample versus the dementia sample are shown in Figures 2–5. Cut-offs and corresponding diagnostic properties for the MoCA-H score with a 2 point adjustment for <12 years of formal education as described by Dawes et al. who studied dementia and cognitively normal participants (Dawes et al., 2023), z-scores and the uncorrected MoCA raw score are displayed in Table 2. The regression analysis showed a significant impact of age (β = −0.45, *p* < 0.001) and of education (β = 0.35, *p* < 0.001), but not of sex (β = 0.01, *p* = 0.81) on the MoCA-H score. Therefore, we studied the impact of age and education by adding 1 or 2 points for ≤12 years of education and/or ≥80 years of age. However, AUC did not significantly improve compared to the unadjusted MoCA score, with AUCs between 0.931 and 0.955.

**TABLE 2:** Diagnostic accuracy and cut-offs for the German MoCA-H for the cognitively normal versus the MCI and the dementia group and for the MCI versus the dementia group.

	Normal cognition versus impaired cognition (MCI + D)	Normal cognition versus MCI	Normal cognition versus dementia	MCI versus dementia
**MoCA-H score after + 2 points adjustment for ≤12 years of formal education**				
AUC	0.952	0.911	0.982	0.839
**Original cutoff** from the English dementia sample (Dawes et al., 2023)	n.d.	n.d.	≤24	n.d.
Correct classification rate	n.d.	n.d.	82.2%	n.d.
Sensitivity	n.d.	n.d.	100.0%	n.d.
Specificity	n.d.	n.d.	64.4%	n.d.
**Balanced cut-off**	≤21	≤22	≤20	≤18
Correct classification rate	88.8%	83.7%	93.0%	78.8%
Sensitivity	87.6%	84.8%	93.5%	76.6%
Specificity	90.0%	82.5%	92.5%	81.0%
**Youden’s Index**	≤21	≤22	≤21	≤18
Correct classification rate	88.8%	83.7%	93.2%	78.8%
Sensitivity	87.6%	84.8%	96.3%	76.6%
Specificity	90.0%	82.5%	90.0%	81.0%
**MoCA-H z-score**				
AUC	0.907	0.838	0.958	0.809
**Balanced cut-off**	−1.24	−1.03	−1.49	−2.11
Correct classification rate	83.9%	79.6%	88.8%	71.5%
Sensitivity	83.9%	79.7%	88.8%	72.0%
Specificity	83.8%	79.4%	88.8%	70.9%
**Youden’s Index**	−1.07	−0.94	−1.45	−2.28
Correct classification rate	84.9%	80.5%	89.9%	75.8%
Sensitivity	89.2%	83.5%	91.6%	69.2%
Specificity	80.6%	77.5%	88.1%	82.3%
**MoCA-H raw value**				
AUC	0.956	0.920	0.983	0.841
**Balanced cut-off**	≤20	≤20	≤18	≤16
Correct classification rate	89.7%	85.9%	93.1%	79.0%
Sensitivity	92.5%	84.8%	92.5%	75.7%
Specificity	86.9%	86.9%	93.7%	82.3%
**Youden’s Index**	≤20	≤20	≤19	≤16
Correct classification rate	89.7%	85.9%	93.8%	79.0%
Sensitivity	92.5%	84.8%	96.3%	75.7%
Specificity	86.9%	86.9%	91.2%	82.3%

Correct classification rate = (sensitivity + specificity)/2. Adjustment for education includes +2 points for ≤12 years of education. n.d. means not done.

A MoCA-H score of ≤21 points was the optimal cut-off for the total group of cognitively impaired (IC sample), when using 2 additional points for ≤12 years of education according to the Balanced cut-off and Youden’s Index with a sensitivity of 87.6% and a specificity of 90.0%. In the MCI group sensitivity was 75.9% at a cut-off of ≤21 and 96.3% in the dementia group. According to the Balanced Cutoff and Youden’s Index, the optimal cut-off to distinguish the MCI group from cognitively healthy participants was ≤22 points, with a sensitivity and specificity of 84.8 and 82.5%, and ≤18 points to distinguish the MCI group from dementia participants with a sensitivity of 76.6% and a specificity of 81%. In the dementia group the optimal cut-off based on the Balanced Cut-off was ≤20 points, whereas the Youden’s Index suggested a cut-off of ≤21. At a cut-off of ≤20 points, sensitivity and specificity were 93.5 and 92.5%, respectively.

### Two separate cut-offs

**FIGURE 6:**
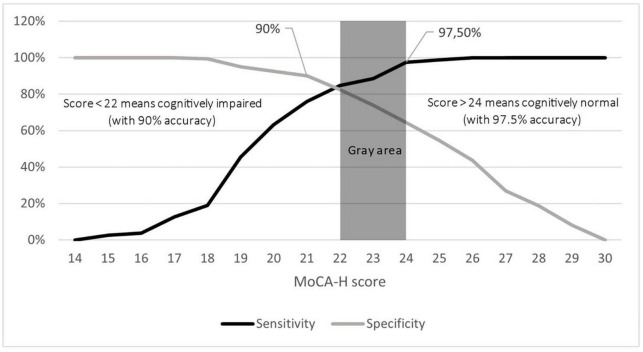
Sensitivity and specificity of the MoCA-H score in MCI. Two cut-offs with a gray area in between in MCI. Sensitivity based on MCI participants (black line) is plotted against specificity based on cognitively normal participants (gray line) after a 2-point-adjustment for ≤12 years of education. The graph displays two cut-offs: the first cut-off (<22) identifies cognitively impaired participants with a specificity of 90%, while the second cut-off distinguishes cognitively normal participants (>24) with a sensitivity of 97.5%. Participants scoring in between these two cut-offs (gray area) require further neurocognitive testing.

There was a large overlap in the MoCA-H score when comparing the MCI and the normal cognitive group. Therefore, it seemed reasonable to define two separate cut-offs (see Figure 6) with an overlap in between as already suggested by Thomann et al. (2020). Sensitivity increased with higher scores, while specificity increased with lower scores. At a cut-off of ≤21 points, specificity was 90%, indicating that only 10% of NC scored lower. At a cut-off point of ≤24 points, sensitivity was 97.5% for the MCI sample. This means, only 2.5% of MCI participants achieved scores higher than 24 points. Hence, a person scoring lower than 21 points most probably is suffering from MCI, while a person who scores higher than 24 points likely has normal cognition.

## Discussion

The present study is the first to establish performance criteria for identifying MCI or dementia among people with and without hearing loss using the German-language MoCA-H.

Recently Dawes et al. validated the English MoCA-H for dementia participants and showed a high sensitivity of 92.8% and a specificity of 90.8% at a cut-off of ≤24 points (Dawes et al., 2023). Using this cut-off point, a high sensitivity of 100% could also be obtained in the German MoCA-H in the present dementia sample. However, specificity was low (64.4%). Studies have shown that optimal cut-offs of the original MoCA vary between different languages (Carson et al., 2018), and cultural or linguistic factors may affect the performance of the MoCA (Ng et al., 2018). Therefore, the performance cut-offs derived for the English MoCA-H may not be optimal for the German translation. Further MCI patients were not included in the English study (Dawes et al., 2023). We consequently developed new cut-off values for the German sample defining cut-offs of the MoCA-H for the detection of dementia as well as for MCI.

Diagnostic accuracy of the German MoCA-H differed depending on the severity of the cognitive impairment. While a cut-off of ≤21 points was considered as optimal in the total cognitive impairment group (IC), showing a sensitivity of 87.6% and a specificity of 90%, sensitivity was significantly lower at this cut-off for the MCI sample (75.9%) and higher for the dementia group (96.3%).

Finding an appropriate cut-off score that can distinguish cognitively healthy individuals from those with mild cognitive impairment is challenging (Carson et al., 2018). While the optimal cut-off for patients with dementia was at ≤21 points in the present study with a sensitivity comparable to the results obtained by the English version, setting a cut-off for patients with MCI was much more difficult. This was already reported by others (Summers and Bondi, 2017; Thomann et al., 2020; Zhuang et al., 2021; Saunders et al., 2022).

Using the optimal cut-off of ≤22 points showed only low sensitivity (84.8%) and specificity (82.5%) in MCI, as there was a large overlap in the MoCA-H score in cognitively healthy and MCI patients. This is in line with a meta-analysis covering 9 studies worldwide on the original MoCA published by Carson et al. (Carson et al., 2018), who found an optimal cut-off of <23 points which strongly deviates from the proposed cut-off of <26 points of the original validation study (Nasreddine et al., 2005) for MCI with a sensitivity of 83% and a specificity of 88%.

To address the issue of diagnostic accuracy, Thomann et al. studying 496 outpatients in the Memory Clinic and 283 normal controls proposed two cut-offs in a range of ≤23 and ≤26 points in the auditory-based original MoCA in MCI (Thomann et al., 2020). This is in line with Yang et al., who questioned whether a single cut-off for the Chinese Beijing version of the MoCA is feasible in order to differentiate the wide spectrum of cognitive disorders and applied a range between >18 and <24 points to achieve a high discrimination rate for the diagnosis of MCI in a large study in 697 Chinese participants aged ≥60 years with a suspicion of cognitive impairment (Yang et al., 2021).

Therefore, we have also developed two cut-offs (>24 points and <22 points) with a range in between. Subjects scoring >24 points are assumed to be cognitively healthy, since at this cut-off point sensitivity is high (97.5%), i.e., only 2.5% of the MCI-subjects score better. Scoring 21 points or less means that there is a high probability that the subject is cognitively impaired, as only 10% of cognitively healthy persons score worse, with a score of ≤18 points indicating dementia and a score between 18 and 21 indicating MCI. In case subjects score in between, a more detailed examination by an experienced psychologist as well as retesting after 6 to 12 months is recommended (Thomann et al., 2020). The approach of using two separate cut-offs with an indecisive area in between might reflect the clinical reality (Saunders et al., 2022) more accurately than a single cut-off (Wong et al., 2015; Thomann et al., 2020; Yang et al., 2021).

Consultation of demographically corrected norms is recommended in the original MoCA to further increase diagnostic accuracy of the test (Nasreddine et al., 2005). In the present study, regression analysis showed a significant impact of age and education on the MoCA-H total score, but adjusting the score by these demographic factors did not significantly change the AUC as already shown by others (Thomann et al., 2020). Whether differences in the educational systems across the countries might account for this discrepancy, remains unclear. However, as sensitivity slightly increased in case education was adjusted for, we decided to adjust as proposed by Dawes et al. (2023). Our findings fit to Thomann et al. (2020), who did not observe a significant improvement in diagnostic accuracy using demographically corrected z-scores in the original MoCA, but reported on differences in the balance of sensitivity and specificity when applying demographic adjustments (Thomann et al., 2020).

Studies on the impact of gender on the original MoCA and the MoCA-H total score are contradictory. While some, including the validation study of the English MoCA-H (Dawes et al., 2023), report about an influence of gender (Konstantopoulos et al., 2016; Borland et al., 2017), the present study found an impact of age and education, but not of gender. This is in line with other normative studies on the original MoCA (Conti et al., 2015; Kopecek et al., 2017).

The presented study is the first on the German MoCA-H in dementia and MCI samples, but it also has some limitations. Although we applied exclusion criteria to exclude individuals with acute infection, delirium, severe psychiatric or brain disorders, we did not perform extensive neuropsychological assessments or magnetic resonance images in all participants. Thereby, the sample of the cognitively unimpaired might also include some people with undetected cognitive dysfunction. However, it should be mentioned that only recruitment of individuals without any kind of abnormal history might result in samples which are not representative of the general population. We believe that the cognitively healthy subjects in the present study are representative for individuals with normal cognitive findings in the clinical routine.

Furthermore, there may be a selection bias in the cognitively normal and the cognitively impaired groups. Whereas the first one was mainly recruited from out-patient settings the MCI and the dementia group consisted of patients attending a geriatric setting. Besides, regional differences in study populations should be taken into account, when applying our findings to other settings. Further, one has to keep in mind that sensitivity, specificity, and the AUC indicate the quality of the test with respect to the reference, but these parameters do not inform about the probability whether a tested person has a specific disease, as predictive values are influenced by prevalence rates. The prevalence rate of dementia and mild cognitive impairment (MCI) in clinical settings beyond geriatric departments might be lower as in the present study where the cognitively impaired patients were recruited in the geriatric clinic.

Ideally, the diagnostic accuracy of a test should be evaluated in the same setting where it is clinically applied (Thomann et al., 2020). Moreover, it is important to note that abnormal scores on a brief neuropsychological screening test like the MoCA-H are insufficient to properly diagnose dementia and require further neuropsychologic evaluation.

## Conclusion

A non-auditory based neurocognitive screening test addresses the issue of over- or misdiagnosis of cognitive impairment in the growing number of hearing-impaired. The German MoCA-H represents a reliable screening tool for MCI and dementia among older people with hearing impairment. In the present study the diagnostic accuracy of the German-language MoCA-H was evaluated in a sample of cognitively healthy individuals as well as in those with mild cognitive impairment (MCI) and dementia. The use of a single cut-off score may be too simple, and a cut-off range should be utilized in MCI diagnostics. However, availability and accuracy regarding biomarkers especially in MCI are still limited (Zhuang et al., 2021). Mixture modeling based on a biopsychosocial perspective and including blood tests, biomarkers, or neuroimaging might improve classification accuracy in the diagnosis of MCI or dementia in the future.

## Data availability statement

The original contributions presented in the study are included in the article/supplementary material, further inquiries can be directed to the corresponding author.

## Ethics statement

The study involving human participants was reviewed and approved by Ruhr-University Bochum, Germany. The patients/participants provided their written informed consent to participate in this study.

## Author contributions

CV and PD designed the study. HF and SF collected the data. HF did the statistical analysis with critical feedback from PD. CV and HF wrote the manuscript with contributions from all other authors. All authors contributed to the article and approved the submitted version.
